# The Expression and Prognostic Impact of CD95 Death Receptor and CD20, CD34 and CD44 Differentiation Markers in Pediatric Acute Lymphoblastic Leukemia

**Published:** 2014-05-21

**Authors:** Fatemeh M. Kamazani, Gholamreza Bahoush-Mehdiabadi, Mahnaz Aghaeipour, Shahram Vaeli, Zahra Amirghofran

**Affiliations:** 1 Department of Immunology, Autoimmune Disease Research Center, Shiraz University of Medical Sciences, Shiraz; 2 Onco-Pathology Research Center, Iran University of Medical Sciences; 3 Research Center of Iranian Blood Transfusion Organization, Tehran, Iran

**Keywords:** Acute Lymphoblastic Leukemia, CD95, CD20, CD34, Prognosis

## Abstract

***Objective:*** This study investigated the expression and prognostic significance of the CD95 death receptor and CD20, a B cell-lineage associated marker, along with CD34 and CD44 non-lineage associated molecules in Iranian children with acute lymphoblastic leukemia (ALL).

***Methods:*** We performed immunophenotyping for expressions of the molecules in blood samples from children diagnosed with ALL by using a panel of monoclonal antibodies for flow cytometry analysis. The expression of markers was evaluated in relation to clinical and paraclinical features as well as response to treatment in the patients.

***Findings*** ***:*** CD95 showed a higher expression in T-ALL compared to B-ALL (*P*<0.001). Analysis of the clinical and laboratory findings at diagnosis in the group of B-ALL patients revealed an association between CD95 expression with lower white blood cell (WBC) numbers and bone marrow blasts (*P*<0.05). We detected a positive correlation between the expressions of CD95 and CD44 (*r*=0.445, *P*<0.01) in B-ALL patients. There was an association between CD20 expression and several poor prognostic factors that included increased extramedullary involvement (EMI) and decreased platelet numbers (*P*<0.008). The mean expression of CD34 in B-ALL was higher than T-ALL (*P*=0.004). At follow-up, complete remission duration (CRD) and survival duration did not significantly differ between patients who were positive or negative for each marker.

***Conclusion:*** Association of the studied molecules with several prognostic factors implies the significance of CD95 molecule as favorable and CD20 as unfavorable prognostic markers for childhood ALL.

## Introduction

Leukemia is a hematopoietic malignancy that results from the clonal proliferation of bone marrow cells with impaired differentiation, regulation, and cell death. The acute leukemias arise from neoplastic transformation of hematopoietic stem cells or progenitors with aberrant differentiation and proliferation^[^^1^^]^. These cells accumulate in the bone marrow and cause suppression of the growth and differentiation of normal blood cells. Acute lymphoblastic leukemia (ALL) comprises a group of genetically different malignant cells derived from B- and T-lymphoid origin^[^^2^^]^. In recent years, new pieces of information obtained through immunophenotyping, cytogenetics, genomic profiling and chemotherapy resistance have contributed to a better understanding of the pathology of this complex disorder and to recognition of subgroups of patients who respond differently to therapy^[^^3^^]^. The majority of patients, both children and adults, with ALL are of B-lymphoid origin. The B lymphoblastic leukemia is classified as precursor B leukemia (B-ALL) since the blast cells are neoplastic counterparts of normal B-cell precursors^[^^2^^]^. Current approaches to risk assessment rely on a number of key clinical and laboratory findings such as age at diagnosis, the initial white blood cell (WBC) numbers, and early treatment response. Generally, children aged 1–9 years are said to have a better outcome than infants and adolescents. WBC number is a risk factor with a continuous variable; increasing numbers confer an inferior outcome^[^^4^^]^. 

 The possible prognostic impact of the expression of various markers has been studied in ALL^[^^2^^,^^4^^,^^5^^]^. CD95 death receptor, CD20 (B-lineage) in addition to CD34 and CD44 non-lineage-associated differentiation markers are surface molecules that have been reported to have a relationship with some clinical and laboratory features of ALL patients at presentation, but the results are conflicting^[^^5^^-^^8^^]^. CD95 (APO-1/Fas), a member of the tumor necrosis factor receptor super family is a transmembrane protein that in a caspase-dependent pathway can induce cell death^[^^9^^]^. CD20 is a small molecule that appears to act as a calcium ion channel and regulates B-cell activation^[^^10^^]^. This molecule is the target of widely used therapeutic monoclonal antibodies of human non-Hodgkin's lymphoma^[^^10^^]^. CD44, a cell surface proteoglycan is widely distributed in different cells and can mediate lymphocyte adhesion to the vascular endothelium and extracellular matrix proteins. This multifunctional adhesion molecule participates in various functions including tumor propagation and metastasis^[^^11^^]^. CD34 is a transmembrane glycoprotein that expresses on undifferentiated hematopoietic stem cells or progenitor cells and remains expressed on early myeloid committed progenitors or lymphoid progenitors during maturation stages in leukemic cells^[^^12^^]^. Several studies have been performed to examine the potential impact of these molecules in predicting treatment outcome^[^^13^^,^^14^^]^. However, to the best of our knowledge, the prognostic value of the expression of these molecules in Iranian patients with ALL is unclear. This study has been performed to investigate the relationship of these lineage- and non-lineage associated markers with clinical and immunophenotypic features as well as response to therapy in Iranian pediatric ALL patients.

## Subjects and Methods

This study included 63 newly diagnosed ALL patients (children under 16 years of age; 32 males, 31 females) with a mean age of 4.86±3.6 years who were admitted to Mahak, Ali-Asghar and Mofid Hospitals (Tehran, Iran) during the years 2008 to 2010. Of patients 58 were B-ALL and 4 T-ALL and, one mix lineage. According to the French-American-British (FAB) classification, most patients had L1 morphology and none had L3 morphology. The laboratory and clinical characteristics recorded at presentation were WBC count, platelet count, hemoglobin (Hb) concentration, percentage of blasts in peripheral blood and bone marrow, and extramedullary involvement (EMI). Data of patients' cytogenetic assays as part of their clinical diagnostic work-up were obtained by medical record review. Induction chemotherapy included vincristine, prednisone, L-asparaginase, and doxorubicin. For intrathecal chemotherapy methotrexate was administered once weekly for six sessions. During 2.5 years of follow-up, the complete remission (CR) rate, CR duration (CRD), and survival duration were recorded. The patients were stratified as high-risk (age: ≤1 and ≥10 years; WBC: ≥50×10^9^/L) and standard-risk (age: >1-9 years; WBC: <50 ×10^9^/L).

 The study was approved by the Shiraz University of Medical Sciences Ethics Committee and informed consent was obtained from the patients or their parents.

 Bone marrow aspirates or peripheral blood from patients was obtained before chemotherapy. Routine immunophenotyping was performed with a panel of fluorescence isothiocyanate (FITC) or phycoerythrin (PE)-conjugated monoclonal anti- bodies (Dako, Glostrup, Denmark) including CD7, CD3, CD5 (for T cells), CD19, CD10, CD20, HLA-DR, CD22, CD79a, IgM (for B cells), CD13, CD33, CD14, CD117 (for myeloid cells), terminal deoxynucleotidyl transferase (TdT) and also CD95, CD34 and CD44. FITC- conjugated antibodies were CD3, CD14, CD19, CD20, CD13, CD33, CD34, and TdT. CD95 and other markers were PE-conjugated. Isotype-matched conjugated mouse monoclonal antibodies were used as negative controls for unspecific staining. The samples were fresh or collected within 24 hours. Samples (100 µL) were incubated with each antibody for 30 min at 4°C and then washed twice with phosphate buffered saline. After lysing red blood cells and fixing the samples, they were analyzed in Partec PAS-II flow cytometer (Partec Instruments, Muenster, Germany) using Partec FloMax software. Blast population was gated for analysis by using CD45 expression versus side scatter. For each marker expression, a 20% threshold was selected to identify positive cases. Based on the expression pattern of TdT, HLA-DR, CD19, CD10 and CD20 the B-ALL patients were subdivided into three subtypes including Pro-B (TdT^+^, HLA-DR^+^, CD19^+^, CD10^-^, CD20^-^), Early Pre-B (TdT^±^, HLA-DR^+^, CD19^+^, CD10^+^, CD20^-^), Pre-B (TdT^-^, HLA-DR^+^, CD19^+^, CD10^+^, CD20^+^)^[^^5^^,^^6^^]^. 

 Statistical analysis was performed using the Statistical Package for Social Sciences (SPSS v. 15.0, Abacus Concepts, Berkeley, CA, USA). Quantitative variables were reported as the mean±standard deviation (SD) according to frequency and percentage values and mean±standard error (SE) for durations. The chi-squared and Fisher's exact tests were used for study of the relationship between markers expression and sex, FAB subtype and EMI. Association between markers expression and parameters such as age, Hb, platelet and WBC counts was analyzed with *t*-tests for independent samples. The nonparametric Mann–Whitney U test was used to compare two independent groups and the Kruskal–Wallis test was used for more than two groups. The correlation between markers among B-ALL patients was examined by calculating Pearson’s correlation coefficient. The overall survival was calculated from the date of entry into the study until the date of death and in patients still alive until the end of follow-up. CRD was calculated from the date complete remission was achieved. Survival and remission durations were analyzed by the Kaplan and Meier method^[^^15^^]^ and in different groups were compared by the log-rank test.

## Findings

**Patient's characteristics**

Table 1 reports patients' detailed characteristics. According to the panel of monoclonal antibodies used, out of 63 childhood ALL cases, 58 (92.1%) were diagnosed as B-ALL and 4 (6.3%) were diagnosed as T-ALL. B-ALL was further subdivided into three distinct subtypes: pro-B-ALL (8.6%), early pre-B-ALL (50%), and pre-B-ALL (41.4%).

 The overall mean CBC parameters at diagnosis have been shown in Table 1. Reports of physical examination revealed the presence of EMI in 56.5% of patients; among which splenomegaly was the most common (51.6%). Of 47 ALL patients who had been subjected to cytogenetic analysis, abnormal karyotypes were found in 17 (36.2%) patients, 7 of whom (14.9%) were TEL/AML1 positive.

**Table1:** Characteristics of patients with ALL and the expression of CD95 and other markers

**Variables**				**Mean (SD) or ** **Frequency**
**Total patients **				63
**Age (yrs)**				4.86 (3.6)
	**1≥ age ≥10**			10
	**1< age <9**			52
**Gender (female/male) **				32/31
**FAB Classification**			**L1**	36 (58)
			**L2**	21 (33.9)
			**Mix L1, L2**	5 (8.1)
**WBC×10** ^9^ **/L**				34 (56.4)
**Hb (g/dL)**				8 (2.2)
**Platelet ×10** ^9^ **/L**				93.4 (105)
**% blast **		**Peripheral blood**		54.3 (26.7)
		**Bone marrow**		75.0 (22.0)
**Patients with EMI**				35 (56.5%)
**Patients with cytogenetics abnormality**				17 (36.2%)
**CR Duration (days)**				640 (34)
**Survival (days) **				706 (25)
**CR time (days)**				20 (9.1)
**Patients with CR **				57 (93.4%)
**Death rate**				8 (12.9%)
**CD20** ** expression**				25.1 (24.3%)
**CD44** ** expression**				24.8 (19.8%)
**CD34** ** expression**				34.6 (31.2%)
**CD95** ** expression**				16.6 (18.9%)

Data represent mean ± SD (standard deviation) according to number (%) and mean ±standard error for durations. ALL: Acute lymphoblastic leukemia; FAB: French-American-British classification of ALL; Hb: Hemoglobin; WBC: White blood cell; EMI: Extramedullary involvement; CR: Complete remission.

The overall survival, CRD and CR time were 706±25 (SE) days (median; 749 days), 640±34 (SE) days (median; 718 days) and 20.2±9.1 days, respectively. CRD in 4 high-risk patients (564±146 days) was shorter than that in 42 standard-risk patients (870±21 days, log rank test; *P*=0.04). Among 61 patients whose data of remission was available, 4 patients (6.6%) failed to achieve CR (CR rate; 93.4%). During the study 8 (12.9%) patients died. 

**Characteristics of B- and T-ALL patients: **A comparison of the clinical and laboratory features of patients diagnosed with T- and B-ALL showed that patients significantly differed in FAB subtypes, WBC number, percentage of blasts in peripheral blood and the presence of EMI. Based on FAB criteria all four T-ALL patients were diagnosed morphologically as L2, whereas this subtype was lower in the B-ALL group (29.3%; *P*=0.01). There was a higher WBC count (*P*<0.001) and higher percentage of blasts (*P*=0.02) in the T-ALL group compared to those with B-ALL. EMI that included hepatomegaly, splenomegaly and lymphadenopathy was observed significantly more in T-ALL patients compared to B-ALL (*P*=0.045).

**Patients with B-ALL subtypes and their characteristics: **The features of B-ALL patients in relation to each subgroup are shown in Table 2. There was a lower WBC number in early pre-B-ALL patients which was not significant (*P*=0.07). Of 24 patients with pre-B-ALL, 17presented with EMI at diagnosis (*P*=0.049). None of the other established prognostic parameters were observed to be significantly associated with B-ALL subtypes. Notable is that the only two patients with CNS involvement who also did not enter CR had pre-B-ALL (*P*<0.2).

**Fig. 1:**
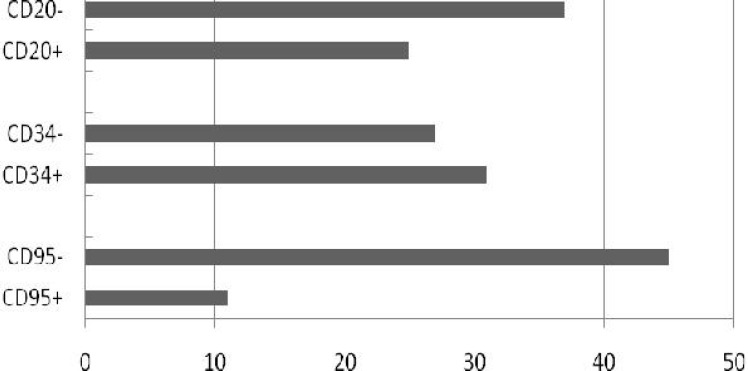
Expression of CD95, CD20 and CD34 in patients with acute lymphoblastic leukemia. For each marker more than 20% expression was considered as positive.

**Expressions of CD95, other lineage and non-lineage associated markers and their relationship with prognostic factors: **A total of 19.6% of all patients were positive for CD95 (Fig. 1). CD95 positivity was observed in 15.4% of B-ALL patients and 75% of T-ALL patients (*P*=0.004). The mean expression of CD95 was higher in T-ALL (49.0±30.6%) than in B-ALL patients (14.1±15.6%; *P*<0.001). There was no significant difference in CD95 expression between different B-ALL subtypes (Table 2).

**Table 2:** Characteristics of patients with various subtypes of B-ALL and the relationship with different prognostic factors, and expression of CD95 and other markers

**Variables**	**ProB-All** **Mean (SD) or ** **Frequency**	**Early preB-All** **Mean (SD) or ** **Frequency**	**PreB-All** **Mean (SD) or ** **Frequency**	***P. *** **value**
**Patients **	5 (8.6%)	29 (50%)	24 (41.4%)	-
**Age (years) **	5.10 (3.51)	5.03 (2.92)	4.60 (3.69)	0.8
**WBC×10** ^9^ **/L**	42.74 (87.92)	13.85 (14.98)	30.65 (28.66)	0.068
**Platelet×10** ^9^ **/L**	156.80 (122.9)	109.69 (126.16)	62.04 (70.36)	0.1
**CRD (days)**	745 (27)	638 (51)	634 (58)	0.6
**Survival (days) **	787.6 (59.4)	672 (45)	725 (33)	0.5
**CR time (days)**	14.00 (0.00)	18.93 (8.9)	20.63 (7.13)	0.3
**% blast (PB)**	85.0 (0.00)	45.00 (28.80	54.24 (22.05)	0.4
**% blast (BM)**	68.82 (28.46)	80.19 (20.67)	69.37 (22.21)	0.1
**Patients with EMI**	1 (3.3%)	13 (41.9%)	17 (54.8%)	0.049
**TEL/AML1 positive**	1 (14.2%)	3 (42.9%)	3 (42.9%)	0.9
**Death rate**	1 (12.5%)	6 (75%)	1 (12.5%)	0.2
**CR rate**	5 (9.3%)	28 (51.9%)	21 (38.8%)	0.6
**CD20 expression (%)**	11.3 (11.3)	9.3 (4.9)	50.18 (20.81)	-
**CD44 expression (%)**	34.21 (23.67)	23.10 (22.86)	25.48 (16.21)	0.5
**CD34 expression (%)**	54.56 (31.82)	29.90 (30.47)	37.82 (31.9)	0.2
**CD95 expression (%)**	21.28 (23.45)	11.89 (11.99)	15.03 (17.41)	0.4

Data represent mean ± SD (standard deviation) according to number (%) and mean±standard error for durations. FAB: French-American-British classification of ALL; PB: peripheral blood; BM: bone marrow; WBC: white blood cell; EMI: extramedullary involvement; CR: complete remission; CRD: complete remission duration

 Analysis of the clinical and laboratory findings at diagnosis revealed that the mean expression of CD95 was higher in patients with Hb<10 g/dL than those with Hb ≥10 g/dL (15.38±17.04 vs 8.87±4.30, *P*=0.03) (Table 3). This molecule had a higher expression in patients with bone marrow blast <50% than patients with bone marrow blast ≥50% (25.80±28.97 vs 11.80±10.11, *P*=0.01). Moreover, the patients with WBC number <50×10^9^/L showed a higher expression of this molecule than those with WBC ≥50×10^9^/L (15.67±16.70 vs 6.76±3.78, *P*=0.003). 

**Fig. 2:**
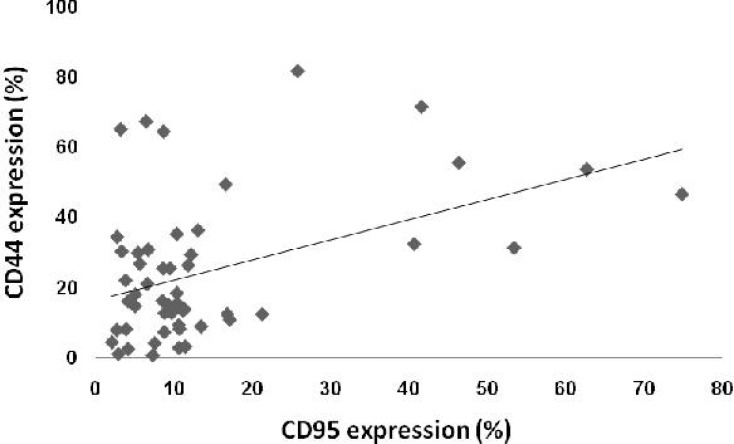
The relationship between CD95 and CD44 expression in patients with B-cell acute lymphoblastic leukemia. A positive correlation between the expression of CD95 and CD44 (*r*=0.445, *P*<0.01) was observed.

 We investigated the relationship between CD95 expression and CD20, an important B-lineage marker, as well as CD34 and CD44 non-lineage associated markers. Our results indicated a positive correlation between the expression of CD95 and CD44 (*r*=0.445, *P*<0.01) in ALL patients (Fig 2). Among 8 ALL patients that were CD95 positive, 7 were also CD44 positive (*P*=0.01).

 CD95 showed no significant correlation with the two other molecules. Data on the expression of other markers in relation to clinical and laboratory features of B-ALL patients are presented in Table 3. CD20 showed a significant relationship with several poor prognostic laboratory features in B-ALL patients. The mean expression of this molecule was higher in patients who presented with EMI (35.11±26.21%) compared to those without EMI (17.21±18.83%, *P*=0.004). This molecule had higher expression in patients with splenomegaly (34.67±27.28%) compared to those without (18.72±19.04%, *P*=0.01) and with CNS involvement (60.35±27.93%) compared to those with no CNS involvement (25.21±23.79%, *P*=0.046). 

**Table 3:** Characteristics of patients with B-ALL and the relationship between CD20, CD34 and CD95 positivity and different prognostic factors

**Variables**			**Total** **n (%)**	**CD20**		**CD34**		**CD95**	
				**Expression %** **Mean (SD)**	***P.*** ** value**	**Expression %** **Mean (SD)**	***P.*** ** value**	**Expression %** **Mean (SD)**	***P.*** ** Value**
**Age **	≤1- ≥10		8	34.5 (32)	0.3	52.4 (25.6)	0.1	19.1 (25)	0.3
**(yrs) **	1-9		50	25.1 (23)		33 (31.6)		13.3 (13.8)	
**Hb (g/dL)**	<10 ≥10		48 (82.8) 10 (17.2)	28.22 (25.77) 17.81 (15.63)	0.09	35.29 (32.01) 36.92 (30.21)	0.8	15.38 (17.04) 8.87 (4.30)	0.03*
**% blast in BM**	<50 ≥50		10 (17.5) 47 (82.5)	29.95 (22.32) 24.72 (24.47)	0.5	44.99 (25.64) 33.33 (32.8)	0.3	25.80 (28.97) 11.80 (10.11)	0.01*
**WBC×** **10** ^9^ **/L**	< 50 ≥50		48 (82.8) 10 (17.2)	24.28 (23.28) 36.72 (28.94)	0.1	34.47 (30.07) 40.99 (39.22)	0.5	15.67 (16.70) 6.76 (3.78)	0.049*
**Platelet ×10** ^9^ **/L **	<100 ≥100		42 (72.4) 16 (27.6)	31.01 (26.24) 15.70 (15.27)	0.008	32.27 (31.21) 44.10 (31.49)	0.2	12.11 (12.30) 20.15 (22.43)	0.2
**Patients ** **with E** **MI**			31 (53.4)	35.11 (26.21)	0.004	28.34 (28.48)	0.3	14.82 (16.62)	0.7
**Without EMI**			27 (46.6)	17.21 (18.83)		43.93 (33.19)		13.25 (14.54)	
**Cytogenetic ** **abnormality **		**Yes**	17 (36.2)	23.05 (25.91)	0.5	32.21 (32.51)	0.8	13.72 (14.42)	0.9
		**No**	30 (63.8)	27.62 (22.99)		34.28 (31.47)		14.03 (16.23)	
**TEL/AML1+** **TEL/AML1-**			7 (14.9)	16.67 (17.32)	0.2	10.98 (12.59)	0.07	7.80 (3.60)	0.2
			40 (85.1)	27.11 (24.99)		36.05 (31.99)		15.06 (16.38)	
**Patients died**			8 (12.9)	15.3 (21.6)	0.1	34.8 (38)	0.9	12.5 (12.1)	0.7
**Alive patients**			50 (87.1)	28.6 (24.7)		35.6 (30)		14.4 (16.3)	

Data represent mean ± SD (standard deviation) and number (%). FAB, French-American-British classification of ALL; Hb: hemoglobin; PB: peripheral blood; BM: bone marrow; WBC: white blood cell; EMI: extramedullary involvement

CD20 expression was higher in patients with platelet numbers less than 100×10^9^/L (30.01±26.24%) compared to other patients (15.70±15.27%, *P*=0.008). The overall expression of CD34 molecule in B-ALL was 35.5±31.4% and for T-ALL it was 22±27.8% (*P*=0.004). More than half of the patients showed 20% positivity for this molecule (Fig. 1). The expression of this molecule in Tel/AML1-positive patients was lower than in patients who were Tel/AML1-negative, however the results were not significant (*P*=0.07). CD34 showed no significant association with the other studied clinical and laboratory features of the patients. CD44 mean expression in patients was 24.8±19.8%. In this study, we have not included the data of the relation of CD44 expression with prognostic factors in B-ALL patients. This data was reported in our previous study with regard to the combined expression of CD27 and CD44 (double positive and/or negative patterns) on this group of patients^[^^12^^]^. The mean CD44 expression was lower in TEL/AML1-positive B-ALL patients compared to TEL/AML-negative patients^[^^16^^]^.

 The mean expression of molecules (except CD20 which was used in categorization) did not differ between B-ALL immunophenotypic subtypes (Table 2).

**Response to therapy: **After 2.5 years of follow-up, we investigated the treatment response in patients in relation to ALL subtypes and the expression of markers.

 According to the data, T-ALL patients had significantly longer CR times (34±23.6 days) compared to B-ALL patients (19.5±7.5, *P*=0.007). Analysis for CRD and survival duration showed no significant difference between the various ALL subtypes. Overall survival as estimated by Kaplan-Meier for patients who had positive expression (>20%) and those who had negative expression (<20%) for each marker showed no significant differences (Table 4). Due to the low number of patients who did not achieve a CR, survival analysis for estimation of CRD in relation to the positivity of the markers was not performed. The results of markers' expressions in relation to CR rate and risk groups showed some significant results; however, due to the low number of patients this data was not reported. None of the studied markers showed an association with death rate.

**Table 4:** Survival analysis in B-ALL patients in relation to expression of CD95, CD20 and CD34 positivity

**Patients**	**Survival (days)** **Mean (SE)**	***P.*** ** value**
**CD20+**	832 (45)	0.1
**CD20-**	827 (33)	
**CD34+**	906 (30)	0.4
**CD34-**	712 (60)	
**CD95+**	805 (81)	0.8
**CD95-**	844 (41)	

SE: standard error. *P* value represents differences between patients who were positive for each marker (>20% expression) and those who were negative (<20%) analyzed by log rank test. No significant differences were observed.

## Discussion

In this study we investigated the prognostic value of several lineage- and non-lineage associated markers in Iranian pediatric ALL patients. Patients included those with both B-ALL and T-ALL immunophenotypic subtypes. Approximately 92% of our patients were B-ALL whereas approximately 6% were T-ALL. In a previous study on Iranian patients there were more T-ALL patients (15%) than seen in the current study however that research included both pediatric and adult patients^[^^17^^]^. 

 The classification into B- and T-ALL is important for risk stratification and treatment. Analysis of the association between clinical and laboratory findings within these subtypes has revealed a significantly longer CR time for T-ALL patients. This group showed relatively more resistance of T cell blasts to chemotherapy. Despite a small population in the T-ALL group, we found higher WBC numbers and percentage of blasts in peripheral blood of these patients. Moreover, EMI was observed more in T-ALL patients compared to B-ALL patients. These data and our results regarding the association of this subtype with L2 morphology were in line with previous studies that reported a poorer prognosis in the T-ALL group compared to the B-ALL group^[^^18^^,^^19^^]^. 

 In this study because of the higher number of B-ALL patients we mainly focused on this group. As our study showed, the majority of these patients were early pre-B-ALL whereas the pro-B-ALL group had the least number of patients. Pro-B-ALL is the most undifferentiated B-ALL and lacks the expression of CD10. It is in close association with the t(4;11) translocation. According to a previous study, this subtype has been recognized to have a poorer prognosis^[^^20^^]^. However, in the present study we did not find any significant differences except for a higher platelet count in this group. A higher platelet count has been suggested as a positive prognostic factor in a previous study on Iranian patients^[^^21^^]^. This difference might be attributed to the small number of cases with this subtype or a variation in the patient groups. 

 The group of patients with CD10 positivity (common ALL) is a large group, which represents the majority of childhood ALL. This group is considered to have a good prognosis; approximately 85% of these children enter long-term remission^[^^22^^]^. This group however is heterogeneous in terms of response to therapy according to cytogenetic and molecular markers^[^^22^^]^. In the present study we did not measure cytoplasmic immunoglobulin, therefore we generally subclassified CD10 positive B-ALL patients according to the expression of CD20 into two groups, CD20 negative (early pre-B) and CD20 positive (pre-B). In a previous study on Iranian patients, the early pre-B group (pre-B I) was the most represented phenotype with a frequency of 51.2% among 43 children with B-ALL which was similar to our results (50%)^[^^23^^]^. According to the results obtained, we found no significant differences between B-ALL subtypes except in relation to EMI which was higher in pre-B-ALL patients. This data was in line with several previous studies that have shown an unfavorable prognosis for the pre-B group^[^^22^^,^^24^^]^. In previous studies the prognostic significance of CD20 expression has been investigated in B-ALL children and adults however it is still a subject of debate. In pediatric ALL, Borowitz et al have found that high CD20 expression correlated with poorer event-free survival^[^^25^^]^, whereas Jeha et al did not report CD20 as an adverse prognostic factor^[^^7^^]^. In a recent study, Mannelli et al failed to demonstrate a prognostic significance for CD20 expression in B-ALL^[^^26^^]^. In our study the mean expression of this molecule was significantly higher in patients who presented with EMI and in those with platelet numbers less than 100×10^9^/L. CD20 expression was higher in patients who did not enter into CR, which showed its relation to the poor prognostic features of ALL.

 In the present study we have investigated expression of the CD95 death receptor and CD34, a non-lineage associated marker in T-ALL and B-ALL, and their relationship with different prognostic factors. CD95 is expressed by many nucleated cells and plays a crucial role in differentiation, regulation and development of myeloid and lymphoid cells^[^^27^^]^. The interaction of CD95 with its ligand transduces an active signal for cellular apoptosis^[^^28^^]^. This interaction is an important mechanism in the destruction of tumor cells by cytotoxic T lymphocytes and natural killer cells^[^^28^^]^. Reduced expression of CD95 can lead to loss of sensitivity to apoptosis that is associated with an increased risk of cancers^[^^29^^]^. In several studies the expressions of apoptotic markers such as CD95 and Bcl-2 have been investigated in cancer patients, including leukemia^[^^29^^-^^33^^]^. Down regulation of CD95 expression in high-risk AML patients as well its alterations in patients with acute promyeloblastic leukemia (APL) under all-trans retinoic acid therapy have been shown^[^^34^^,^^35^^]^. Examination of CD95 expression in primary human acute leukemic cells by flow cytometry has shown quantitative differences between different forms of acute leukemia, such that T-ALL blasts had greater expression of CD95 compared to B-ALL blasts^[^^36^^]^. Similarly, in our study the proportion of CD95 positive cases was greater in T-ALL than in B-ALL patients. The mean expression of CD95 was also higher in the former group. In this study there was a significant negative correlation of CD95 expression with Hb, WBC number and percentage of blasts in bone marrow which suggested that this molecule was a favorable prognostic factor. In a previous study, high-level CD95 expression predicted a favorable response to chemotherapy in ALL^[^^37^^]^. Chemotherapeutic agents have been shown to promote the apoptotic death of leukemic cells by up-regulating CD95 expression levels^[^^38^^]^.

 We also found a significant positive correlation between CD95 and CD44 expression which, to the best of our knowledge, has not previously been reported in ALL. CD44 was proposed to be involved in the induction of Fas expression and the subsequent augmentation of Fas-mediated apoptosis in synovial cells^[^^39^^]^. Moreover, deficiency in CD44 in combination with a defect in Fas has been shown to down-regulate activation-induced cell death and increase lymphoproliferative diseases in mice^[^^40^^]^. The mechanism of association of CD44 and CD95 in ALL needs to be further investigated.

 With respect to CD34, the prognostic significance of this molecule's expression has shown conflicting results, and appears to be dependent on the types and subtypes of leukemias and the treatment regimen used^[^^6^^,^^9^^]^. Previous studies have proposed that CD34 expression is a good prognostic factor in childhood ALL^[^^6^^]^. In our study this molecule has shown no significant correlation with studied prognostic factors such as WBC numbers, EMI and survival duration. As previously mentioned, the low number of patients who did not achieve CR did not allow us to undertake a reliable analysis for the expression of the studied markers in relation to CR rate and duration as important indicators of response to therapy. A significant finding in our study in relation to CD34 was its higher expression in B-ALL compared to T-ALL, which has been reported previously^[^^6^^]^.

## Conclusion

Evaluation of the expression of several markers with established prognostic factors and response to therapy in a group of Iranian childhood ALL patients has shown a positive impact for CD95 expression and a negative impact for CD20 expression in these patients. This result emphasizes the importance of evaluating these markers as prognostic factors in pediatric ALL in future larger cohorts.
